# Biotransformation of Chloroaromatics: the Impact of Bioavailability and Substrate Specificity

**DOI:** 10.1155/S1565363304000135

**Published:** 2004

**Authors:** Demetrio Randazzo, Marta Ferraroni, Andrea Scozzafava, Ludmila Golovleva, Fabrizio Briganti

**Affiliations:** 1 Laboratorio di Chimica Bioinorganica Dipartimento di Chimica Università di Firenze Via Della Lastruccia 3 Firenze 50019 Italy; 2 Skryabin Institute of Biochemistry and Physiology of Microorganisms Pushchino Moscow region RAS 142290 Russia

## Abstract

The effect of surfactants on the biodegradation of mono-aromatic hydrocarbons such as benzene, chlorobenzene and 1,2-dichlorobenzene by an Escherichia coli JMI09(MI) recombinant strain, carrying a gene cluster containing the genes for benzene dioxygenase, cis-benzene dihydrodiol dehydrogenase, and catechol 2,3-dioxygenase from Pseudomonas putida ML2, has been investigated. We observed that the efficiency of the benzene dioxygenase catalyzed conversions to cis-dihydrodiols depends on the balance among real substrate specificity, bioavailability, and toxicity effects of highly concentrated aromatic hydrocarbons. The utilization of non ionic surfactants makes it possible to partly overcome the limiting step of biodegradation processes for scarcely water soluble hydrocarbons hindered by their limited bioavailability.

Furthermore the cis-benzene dihydrodiol dehydrogenase and the extradiol catechol 2,3-dioxygenase, which in the presently analyzed biodegradative pathway should further degrade the pollutants, are known, the first to be selectively specific for the (lR,2R)-dihydrodiol derivative which is not produced by the benzene dioxygenase, the second, to be dead-end inhibited by the corresponding chlorinated catechois. In the present example this results in the accumulation of the corresponding chlorinated cis-dihydrodiols which can be useful for asymmetric synthesis.

On the other hand the practical utilization of the system for bioremediation purposes requires the efficient conversion of the chlorinated catechols by specific intradiol ring-cleaving dioxygenases, the crystal structures of some of these last enzymes are currently under analysis in our laboratory to understand the structuralfunctional correlations. Preliminary data show overall structures similar to the catechol 1,2-dioxygenase from Acinetobacter sp. ADP1 thus suggesting that the substrate specificity differences are mainly related to subtle differences in the catalytic site.


### Biotransformation of Chloroaromatics: the Impact of Bioavailability and Substrate Specificity

Demetrio Randazzo[1], Marta Ferraroni[1], Andrea Scozzafava[1],

Ludmila Golovleva[2], Fabrizio Briganti[1,*]

[1]*Laboratorio di Chimica Bioinorganica, Dipartimento di Chimica, Università di Firenze*
[2]*Skryabin Institute of Biochemistry and Physiology of Microorganisms RAS 142290, Pushchino, Moscow region, Russia*

### ABSTRACT

The effect of surfactants on the biodegradation of mono-aromatic hydrocarbons such as benzene, chlorobenzene and 1,2-dichlorobenzene by an *Escherichia coli* JM109(M1) recombinant strain, carrying a gene cluster containing the genes for benzene dioxygenase, cis-benzene dihydrodiol dehydrogenase, and catechol 2,3-dioxygenase from *Pseudomonas putida* ML2, has been investigated.

We observed that the efficiency of the benzene dioxygenase catalyzed conversions to cis-dihydrodiols depends on the balance among real substrate specificity, bioavailability, and toxicity effects of highly concentrated aromatic hydrocarbons. The utilization of non ionic surfactants makes it possible to partly overcome the limiting step of biodegradation processes for scarcely water soluble hydrocarbons hindered by their limited bioavailability.

Furthermore the cis-benzene dihydrodiol dehydrogenase and the extradiol catechol 2,3-dioxygenase, which in the presently analyzed biodegradative pathway should further degrade the pollutants, are known, the first to be selectively specific for the (1R,2R)-dihydrodiol derivative which is not produced by the benzene dioxygenase, the second, to be dead-end inhibited by the corresponding chlorinated catechols. In the present example this results in the accumulation of the corresponding chlorinated cis-dihydrodiols which can be useful for asymmetric synthesis.

On the other hand the practical utilization of the system for bioremediation purposes requires the efficient

* To whom correspondence should be addressed:
Prof. Dr. Fabrizio Briganti
Laboratorio di Chimica Bioinorganica,
Dipartimento di Chimica, Università degli Studi di Firenze,
Via Della Lastruccia 3, 50019, Firenze, Italia
Phone (+39)0554573343; FAX (+39)0554573333;
E-mail: fabrizio.briganti@unifi.it conversion of the chlorinated catechols by specific intradiol ring-cleaving dioxygenases, the crystal structures of some of these last enzymes are currently under analysis in our laboratory to understand the structural-functional correlations. Preliminary data show overall structures similar to the catechol 1,2-dioxygenase from *Acinetobacter* sp. ADP1 thus suggesting that the substrate specificity differences are mainly related to subtle differences in the catalytic site.

**Keywords:** Benzene, Chlorobenzene, 1,2-dichlorobenzene, Benzene dioxygenase, Surfactants, Direct micellar systems, Biodegradation, Biotransformation.

### INTRODUCTION

Remediative or synthetic technologies based on microbial transformations of hydrocarbons (biotransformations hereafter) are becoming every day more relevant. In particular in situ processes for the removal of polluting aromatic hydrocarbons can potentially benefit of the capabilities of enzymatic systems in terms of faster and gentle treatment conditions, reduced environmental pollution, and low costs /1,2/. Furthermore environmentally benign procedures utilizing such enzymes have interesting applications for the synthesis of useful chemicals /3-7/.

The aerobic biotransformation of aromatic hydrocarbons always require the action of oxygenases, often containing iron ions essential for the catalysis of the aromatic ring hydroxylation and the consecutive ring opening /8,9/. In bioproduction processes however the use of isolated enzymes is not realistic mainly because hydroxylating oxygenases are unstable multi-component systems difficult to purify and requiring expensive co-substrates /10,11/. Therefore technologies using whole cells engineered microorganisms which provide for the over-expression of such enzymatic systems have been developed /12, 13/.

*Pseudomonas putida* ML2, a microorganism first described in 1973, is able to utilize benzene as the sole carbon and energy source /14/. This bacterium oxidizes benzene to cis-1,2-dihydroxy-cyclohexa-3,5-diene (cis-benzene dihydrodiol) through the addition of both atoms of dioxygen to the aromatic nucleus (reaction catalyzed by benzene 1,2-dioxygenase, (BDO)). Afterwards the product is oxidized to catechol by a cis-dihydrodiol dehydrogenase (BCD). Finally catechol is further converted through oxygenative ring cleaving by catechol 2,3-dioxygenase (C2,3O) to α-hydroxymuconic semialdehyde which after a few reactions enters the regular carbon cycle (see Scheme 1) /15-18/.

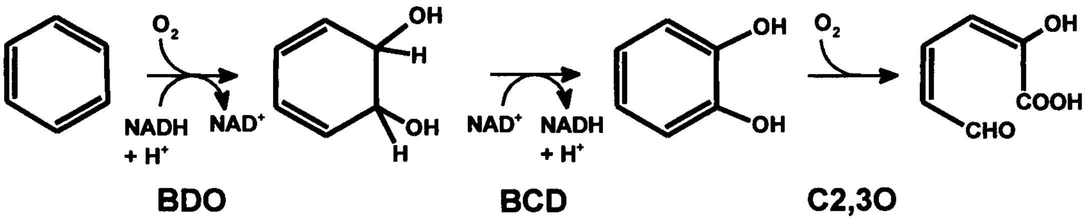

Scheme 1

One of the main drawbacks in biotransformation processes of aromatic hydrocarbons is generally the very low rate of catalysis due to a variety of factors such as the scarce mass transfer rate of the hydrophobic substrates, the restricted enzymatic substrate specificity, and the toxicity effects exerted by such compounds on micro-organisms /12/.

In the present work we will mainly focus on bioavailability limitations to efficient biotransformation procedures, briefly mentioning the aspect of enzymatic specificity for chlorobenzenes.

Regarding bioavailability two-phase systems (like water/organic solvents) in which the major component is an organic solvent able to solubilize large quantities of the hydrophobic pollutants have been formulated, the major drawback being that frequently the cell viability is fairly reduced after only few hours due to the toxic effects of the organic phase /19,20].

Living systems accomplish high levels of selectivity and efficiency using compartmentalization through self assembling systems often composed by amphiphatic units similar to those of synthetic surfactants which form micellar systems above the critical micellar concentration /21-23/. Direct micelles are capable to solubilize amounts of apolar compounds in their core /24-26/. Such systems can be regarded as chemical microreactors, where hydrophobic substances stored in the apolar center can diffuse to the hydrophilic aqueous medium or directly to the adjoining cell membranes /27/.

Micellar phases in microbial bioconversions have been observed to be milder than vigorous mixing of multiphasic media, and the effective improvement of conversion kinetics has been investigated into details for polyaromatic hydrocarbons /28-31/. In such cases high substrate conversion rates and yields were observed together with high cell viabilities.

Regarding enzyme specificity we can remark that the removal of contaminant mixtures from polluted environments is particularly challenging. In fact mixtures of halogenated and alkylated aromatic hydrocarbons are widely spread pollutants and generally the degradation pathways for haloaromatics differ considerably from those of the alkyl-substituted analogues. In particular the ring cleaving dioxygenases involved in the transformation of alkyl-aromatics are generally of the extradiol type whereas those implicated in the cleavage of halogenated aromatics belong to the intradiol class. Strict substrate specificities are also observed among enzymes belonging to the same class of dioxygenases. Only one exception of extradiol cleaving dioxygenase able to catalize the cleavage of haloaromatics has been recently observed /32-34/.

The understanding of the structural factors which cause the observed enzymatic specificities has to be regarded as crucial for the design of more efficient bioremediation techniques.

In the present paper the influence of bioavailability on the efficiency of biotransformation processes for aromatic hydrocarbons like benzene and chloroaromatics like chlorobenzene and 1,2-dichlorobenzene is examined.

The data clearly show reduced affinities of the benzene dioxygenase for the chlorinated compounds although the presence of micellar systems contributes to increase the transformation rates and yields. Anyway the surfactant does not allow to change the real substrate specificities and to prevent the inactivation of the extradiol ring cleaving dioxygenases. It is known that the normal intradiol catechol dioxygenases are unable to cleave chlorocatechols; therefore we have started to determine the x-ray structures of some intradiol dioxygenases specializing in the cleavage of 4-chlorocatechol and hydroxyquinol to reveal the factors essential for substrate selectivity.

### EXPERIMENTAL

### Materials

Triton X100, benzene, chlorobenzene and 1,2-chlorobenzene, were purchased from Sigma-Aldrich S.r.l. (Milan, Italy). Water was purified in a reverse osmosis Milli-Ro system and subsequently treated with a Milli-Q system (Millipore). All the other chemicals were of the best purity available.

### Microorganisms

The *E. coli* JM109(M1) recombinant strain carrying benzene dioxygenase where DNA shuffling was applied to bedC1 gene (reductase component), cis-benzene dihydrodiol dehydrogenase, and the catechol 2,3-dioxygenase cloned from *Pseudomonas putida* ML2 was used for the biodegradation studies and kindly supplied by Dr. J.R. Mason (King's College, London, UK). Such a biological system is able to catalyze the conversion of benzene and other aromatic compounds to the corresponding semialdehydes as previously reported /14-16/.

### Culture media and conditions

*E. coli* inocula were initially grown in LB medium plus ampicillin 100 μg/ml for 8-12 hours then transferred to M9 medium additioned with succinate 20 mM and the appropriate surfactant under standing or stirring conditions at 30°C /35/. The expression of the dioxygenases cluster was constitutive.

Viability experiments were performed by plating estimated amounts of cells, obtained after dilution with LB medium, on LB-agar plates. After 12-18 hours incubation at 30°C the grown colonies, corresponding to the viable cells, were manually counted.

Bioconversion experiments were performed at 30°C, 100-200 rpm orbital shaking, adding to fresh M9 medium with the appropriate concentrations of surfactant, oil, hydrocarbon, and $1.6 \times 10^9$ cells/ml corresponding to about 60-80 Units/liter of benzene dioxygenase activity.

One unit of dioxygenase activity is defined as the amount of cells producing 1 μmol of 1,2-dihydro-1,2-dihydroxybenzene in 1 min under the test conditions.

### HPLC determination of bioconversions kinetics

Reverse phase C18 columns (4.6 x 150 mm) connected to a Waters HPLC Alliance 2690 and a Waters 996 photodiode array detector (Milford, MA, U.S.A.) both interfaced to personal computers were used under isocratic flows (55 % methanol, 45 % water supplemented with 1 g/l $H_3PO_4$) of 1 ml/min for the analysis of the reaction mixtures. 5-20 μl samples were injected after 3' centrifugation at 14,000 rpm and 4°C. The absorbance of the eluate was monitored between 200 and 600 nm. The concentrations of compounds at different times during the kinetic runs have been determined from the areas of the corresponding peaks. All experiments were carried out in duplicate and the values reported are means.

### Analysis of Bioconversion Products

The products generated by the bioconversions of benzene, toluene and ethylbenzene catalyzed by the *E. coli* JM109(M1) recombinant strain carrying the genes cluster cloned from *P. putida ML2* were analyzed using LC-MS and UV-Vis spectroscopy techniques.

For the liquid chromatography-mass (LC-MS) experiments, a Micromass/Waters ZMD 2000 quadrupole system, connected to a Waters Alliance 2690 HPLC was used (Milford, MA, U.S.A.). The mass spectrometer was calibrated using $NaCsI_2$. A mass range of 20-300 amu was scanned and the data were acquired with the MassLynx NT 3.5 data system.

### UV-Vis Spectroscopy

UV measurements were carried out on double beam Varian Cary 3 spectrophotometers using 1.0-0.1 cm path length Hellma 110 quartz suprasil cells.

### RESULTS AND DISCUSSION

The cluster of enzymes: benzene dioxygenase, cis-benzene dihydrodiol dehydrogenase, and the catechol 2,3-dioxygenase cloned from *Pseudomonas putida* ML2, utilized in the present investigation, is known to convert benzene and, with lower efficiencies, other substituted monocyclic aromatic hydrocarbons to the corresponding semialdehydes /14-16/.

To increase the efficiency of the system under investigation DNA shuffling experiments on the bedC1 gene (reductase component) of BDO made it possible to obtain the present clone which shows an enhanced catalytic activity towards benzene conversion to the corresponding semialdehyde (250-300 % enhancement was observed with respect to the native system) (personal communication, Dr. J.R. Mason, King's College, London, UK) /14-16/.

With the exception of benzene, the solubility of which is around 23 mM in water systems at 20°C, the limited solubilities of the other substrates tested in water media (see Table 1) could not allow optimal conversion rates. Possible approaches to more efficient biotransformation processes could employ a two phase system but the contact area between the two phases which should influence the substrate transfer rate is scarce even under vigorous stirring /19, 20/. A much higher contact surface can be obtained in micro-compartmentalized media, where the hydrophobic substrates are dispersed down to nanometer sized droplets stabilized by a surfactant monolayer.

**Table 1**

Solubilities for the different aromatic compounds in the media tested in the present work

| Substrate | Solubility in Water | Solubility in M9/TritonX100 1.5% |
|---|---|---|
| **Benzene** | $2.24x10^{-2}M$ | $> 5.0x10^{-2}M$ |
| **chloro-benzene** | $4.14x10^{-3}M$ | $> 1.0x10^{-2}M$ |
| **1,2-dichloro-benzene** | $6.80x10^{-4}M$ | $> 0.5x10^{-2}M$ |

Dilute direct micellar systems made using suitable non-ionic surfactants can increase the hydrophobic substrate solubility and provide a less aggressive environment for bacterial cells /27-31/. For this reason we utilized 1.5% v/v of non-ionic Triton X100® (t-octylphenoxypolyethoxyethanol) in the mineral culture medium (M9) for the *E. coli* strain containing the genes for the overexpression of the benzene dioxygenase cluster (see Experimental).

A general property of $L_1$ micellar solutions is their ability to solubilize a certain amount of hydrophobic additives. It has been reported that the solute localization in the micellar micro-compartment is directly related to its hydrophobicity, so that the more polar or polarizable compounds such as aromatic hydrocarbons are preferentially found in the so called palisade layer where they interact with the polar group /21, 22/.

Previous studies on microemulsion systems showed that higher apparent solubilities for polyaromatic hydrocarbons provided higher conversion rates and yields /28-31/.

The bacterial strain viabilities in selected surfactant modified media were tested under stirring and standing conditions and provided evidence of optimal growth environments /28].

As expected on the basis of benzene water solubility, the addition of Triton X100 1.5% to the bioconversion environment for such substrate did not provide any further improvement in mass transfer rates and therefore the conversion rates and yields were not enhanced (data not shown).

In such micellar solutions it was possible to raise the real benzene concentration even above 50 mM but increasing the substrate concentration from 10 to 20 and to 40 mM resulted in substantial reductions of the conversion rates due to toxicity problems (see Figure 1). It is known that the addition of benzene and other mono-aromatic hydrocarbons to the culture medium yields the accumulation of such compounds in the hydrophobic segment of the bacterial membrane causing an increased permeability of the cytoplasmic membrane and resulting in impaired bacterial growth due to metabolic energy losses and macromolecules leakage /36-38/. Consequently the final conversion yield that for 10 mM (and lower concentrations) benzene concentration was 100% was reduced to about 80 % and 65 % for 20 and 40 mM starting benzene concentration respectively.

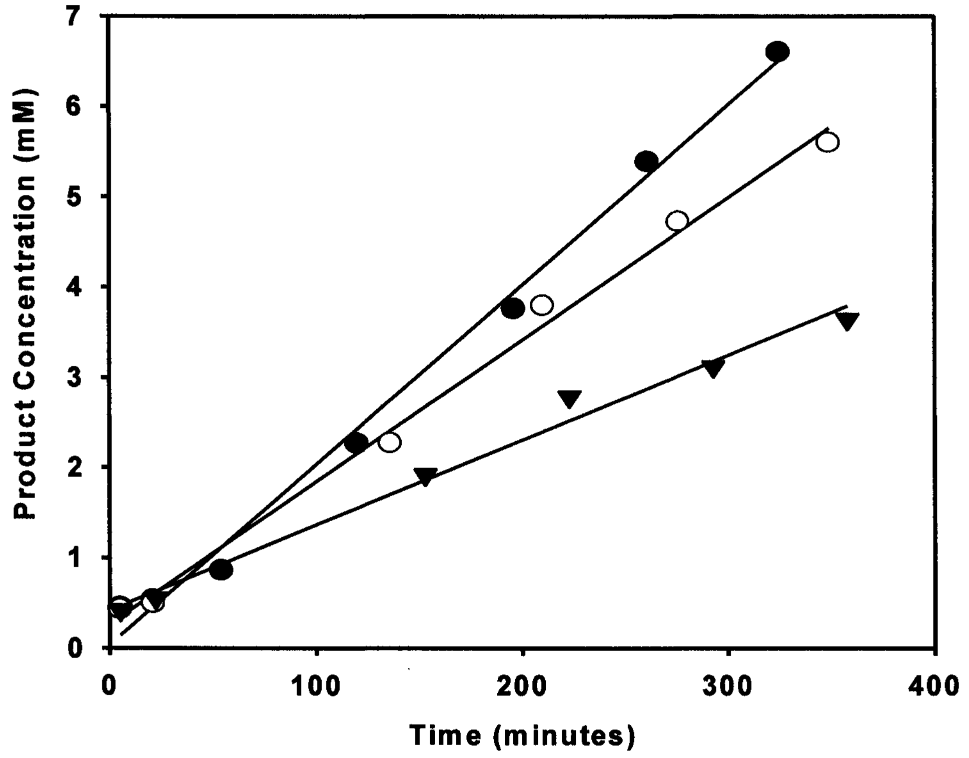

**Fig. 1:** Time-dependent bioconversions of different concentrations of Benzene in micellar systems of 1.5% v/v Triton X100 in M9 (starting concentrations 10mM (●), 20 mM (O), or 40 mM (▼) benzene). The final cell concentration of the induced *E. coli* JM109(M1) recombinant strain resulted to be 1.6 x $10^9$ cells/ml. The kinetics were run at 30°C and 140 rpm.

In order to prevent metabolic energy losses we tried to further add 1 mM NADH or 10 mM ethanol to the culture medium but this did not show any appreciable effect on the conversion process rates and final yields.

The conversion kinetics for chlorobenzene in M9 minimal medium and Triton X100/M9 solutions (1.5% w/w in surfactant) are illustrated in Figure 2. Contrarily to what was observed for benzene, the addition of the non ionic surfactant produces enhancements of the overall conversion rates at 5 mM initial substrate concentration and a four-fold increase of the final yield. As in the case of benzene the doubling of the initial concentration for the aromatic hydrocarbon (in this case from 5 to 10 mM) causes substantial reductions of the catalytic rates due to the toxic effects of such substrate (data not shown).

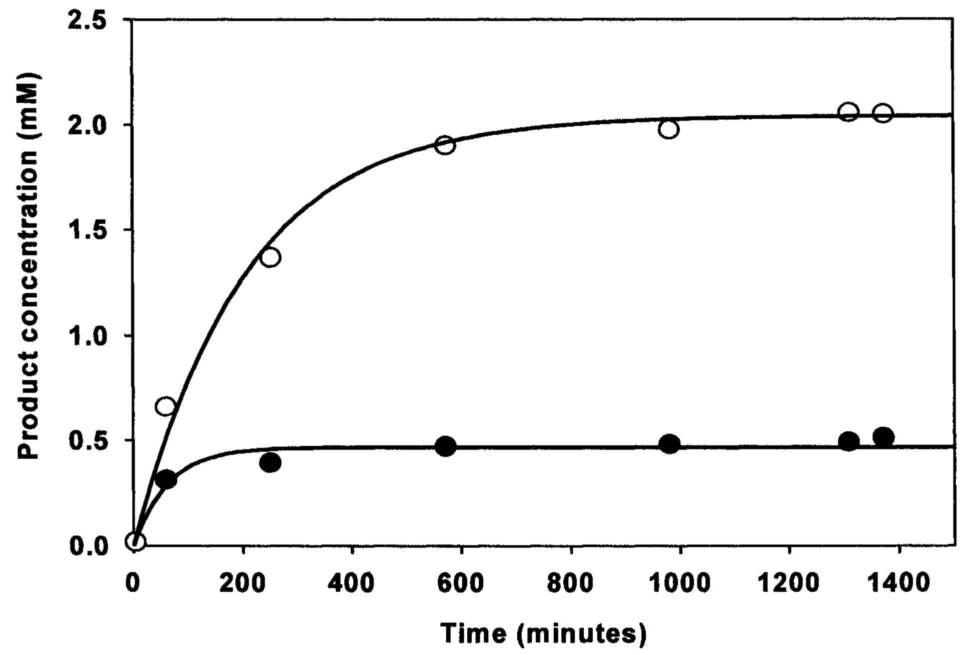

**Fig. 2:** Time-dependent bioconversions of 5 mM chlorobenzene in acqueous M9 (●) or in micellar systems containing of 1.5% v/v Triton X100 in M9 (O). The final cell concentration of the induced *E. coli* JM109(M1) recombinant strain resulted to be 1.6 x $10^9$ cells/ml. The kinetics were run at 30°C and 160 rpm.

LC-MS experiments allowed us to confirm and quantify the products of chlorobenzene conversion. As observed in scheme II the action of benzene 1,2-dioxygenase on chlorobenzene results mainly in the formation of 1S,2S-3-chloro-cis-1,2-dihydroxy-1,2-dihydrocyclohexa-3,5-diene which is not further converted into the corresponding catechol or semialdehyde due to the specificity limitations of the corresponding cis-benzene dihydrodiol dehydrogenase, and catechol 2,3-dioxygenase from *Pseudomonas putida* ML2 /39,40]. In fact a recent study showed that the presently investigated cis-benzene dihydrodiol dehydrogenase is not able to convert (1S,2S)-3-chloro-cis-1,2-dihydroxy-1,2-dihydrocyclohexa-3,5-diene to the resulting catechol since it has a selective preference for the (1R,2R) enantiomer which is not produced by BDO /41/.

The formation of traces (<0.2%) of 2-chloro-phenol probably a decomposition product of the starting diol has also been observed. In table 2 are reported the results of products quantification for chlorobenzene conversion in the different media.

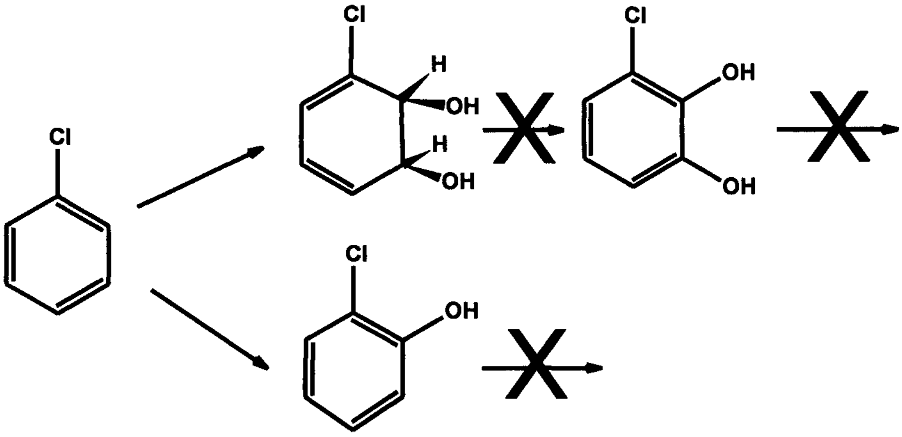

**Scheme II**

Similar results were obtained analyzing the 1,2-dichlorobenzene conversion. In Figure 3 are reported the conversion kinetics for 1,2-dichlorobenzene at 5 mM initial concentration with or without the addition of TritonX100 1.5% w/w to the M9 medium. The addition of the surfactant supports a more efficient conversion of 1,2-dichlorobenzene due to the increased substrate bioavailability, resulting in a six-fold final yield increase.

Also in the case of 1,2-dichlorobenzene the substrate specificity of benzene 1,2-dioxygenase drives towards the formation of 3,4-dichloro-cis-1,2-dihydroxy-1,2-dihydrocyclohexa-3,5-diene that accumulates in the reaction environment (Table2) /42/:

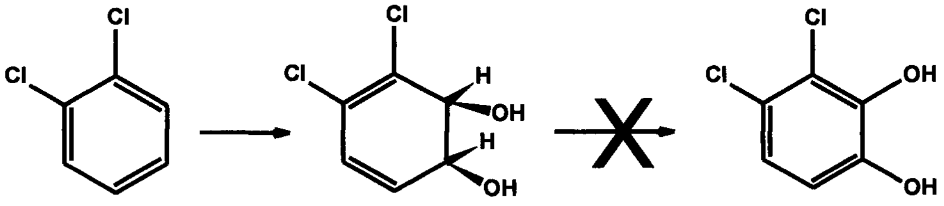

**Scheme III**

Microemulsions obtained by 1% ethyl oleate addition to the micellar systems which had been previously utilized to further increase substrate bioavailability did not shown any additional enhancement in the conversion rates and yields of 1,2-dichlorobenzene (Figure 3, Table 2) /28-30/.

In conclusion the utilization of direct micellar systems able to solubilize aromatic hydrocarbons at high concentrations can allow improvement to a certain extent of the efficiency of biotransformation processes by increasing substrate transfer rates. The present study has also allowed it to be determined that the apparent lower affinity of BDO for chloro-benzene and 1,2-dichloro-benzene than for benzene can be partly overcome in micellar systems where the utilization of suitable surfactants can considerably improve the mass transfer rates and consequently the final product yields.

Furthermore the stringent selectivities of BCD and C2,3O cause the accumulation of significant amounts of interesting chiral synthons that are known to be useful for fine chemicals production /3-7,43/.

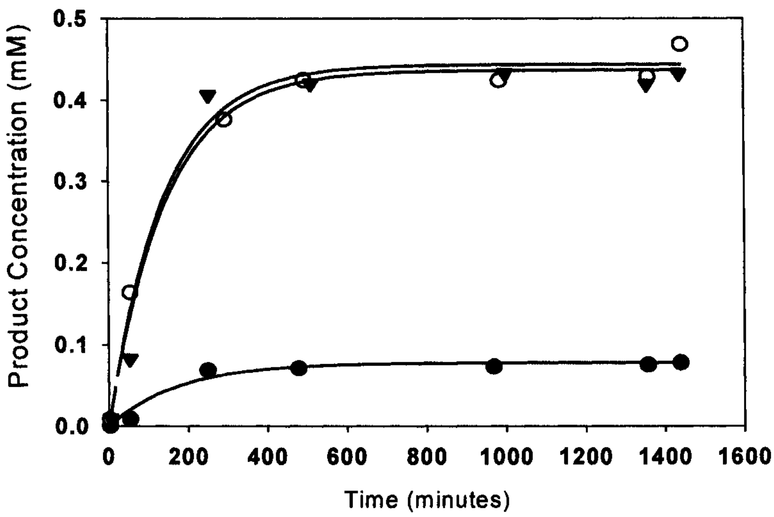

**Fig. 3:** Time-dependent bioconversions of 5 mM 1,2-dichlorobenzene in acqueous M9 (●) or in micellar systems containing of 1.5% v/v Triton X100 in M9 (O) or 1.5% v/v Triton X100 /1% Ethyl Oleate in M9 (▼). The final cell concentration of the induced *E. coli* JM109(M1) recombinant strain resulted to be 1.6 x $10^9$ cells/ml. The kinetics were run at 30°C and 160 rpm.

**Table 2**

Products obtained from chlorobenzene and 1,2-dichlorobenzene biotransformations by the BDO enzymes cluster in different culture media. (Starting substrate concentration 5 mM)

| SUBSTRATES | MEDIUM | PRODUCTS YIELDS | |
|---|---|---|---|
| chloro-benzene | M9 | 10.2% | 3-chloro-cis-1,2-dihydroxy-1,2-dihydrocyclohexa-3,5-diene |
| | M9/ TritonX100 1,5% | 41.2% | |
| 1,2-dichloro-benzene | M9 | 1.5% | 3,4-dichloro-cis-1,2-dihydroxy-1,2-dihydrocyclohexa-3,5-diene |
| | M9/ TritonX100 1,5% | 9.3% | |
| | M9/ TritonX100 1,5%/Ethyl Oleate 1% | 8.6% | |

The formation and accumulation of intermediates during the biodegradation of aromatic hydrocarbons also have implications in the treatment of contaminated waters and gases. In fact many of the observed intermediates are toxic to humans and other organisms in the environment and furthermore such catabolites contain most of the initial COD (chemical oxygen demand) and all of the dissolved organic carbon (DOC) of the original compounds. Therefore the treatment of the aromatic hydrocarbons contamination results to be successful only when the intermediates are also removed /44,45/.

The limitations in biodegradative capacities of different bacteria are mainly due to the presence and nature of the substituents on the dihydroxybenzene ring. Whereas the unsubstituted catechol can be transformed via all pathways, methylated catechols derived from most natural and non-halogenated compounds are assimilated by the meta-cleavage pathway only /46,47/. Unfortunately meta-cleavage dioxygenases as the above reported C2,3O from *P. putida* ML2 are rapidly inactivated by chlorocatechols due to the formation of reactive acyl chlorides; therefore such classes of compounds are typically catabolized via the modified ortho-cleavage pathway which on the other hand is not suitable for methyl-substituted aromatics due to the formation of dead-end methyl-lactones /39,40,48,49/. The simultaneous biodegradation of chloro- and methyl-substituted aromatics often present as mixtures at contaminated sites is possible only by particular bacterial consortia or of particular bacterial strains such as the *P. putida* GJ31 which contains an unusual meta-cleavage dioxygenase able to convert chlorocatechols /32-34].

As mentioned above, intradiol dioxygenases have crucial roles in the modified ortho-cleavage pathway for the biotransformation of halogenated aromatics. Until now only the X-ray structures of a few intradiol dioxygenases have been determined showing large differences in oligomeric structures and sequence homologies /50-55/. These data, together with the information obtained through XAS experiments, are not sufficient to establish the roles of the active site residues in substrate selectivity /56/.

X-ray diffraction studies are in progress in our laboratory for a number of intradiol dioxygenases involved in halogenated aromatic catabolism with different substrate specificities: the hydroxyquinol 1,2-dioxygenase from *Nocardioides simplex* 3E, the 4-chlorocatechol 1,2-dioxygenase and the 3-chlorocatechol 1,2-dioxygenase both from *Rhodococcus opacus* 1CP /57-59/. In particular studies on the two chlorocatechol 1,2-dioxygenases involved respectively in the degradation pathways for 4-chlorophenol (4-chlorocatechol 1,2-dioxygenase) and 2-chlorophenol (3-chlorocatechol 1,2-dioxygenase) from *Rhodococcus opacus* 1CP ascertained that these enzymes differ significantly in their catalytic properties /60,61/.

Our preliminary analysis of the x-ray data shows that the overall crystal structures of these enzymes are very similar to that of the catechol 1,2-dioxygenase from *Acinetobacter Sp.* ADP1 in terms of quaternary structure arrangements, molecular domains and active site positioning (see Figure 4 for hydroxyquinol 1,2-dioxygenase from *Nocardioides simplex* 3E, and 4-chlorocatechol 1,2-dioxygenase from *Rhodococcus opacus* 1CP) /62/. The refined three-dimensional structures of these enzymes with different substrate specificities will provide details on the influence of the active site conformation and aminoacid substitutions involved in substrate selectivity and it will also further improve the knowledge of the catalytic mechanism of this class of dioxygenases.

Only a multidisciplinary approach investigating substrate bioavailability, enzyme specificity and efficiency and utilizing a variety of techniques such as molecular biotechnology, enzyme structure and mechanism characterizations will allow to positively answer to the everyday more demanding requests of bioremediative and biosynthetic technologies.

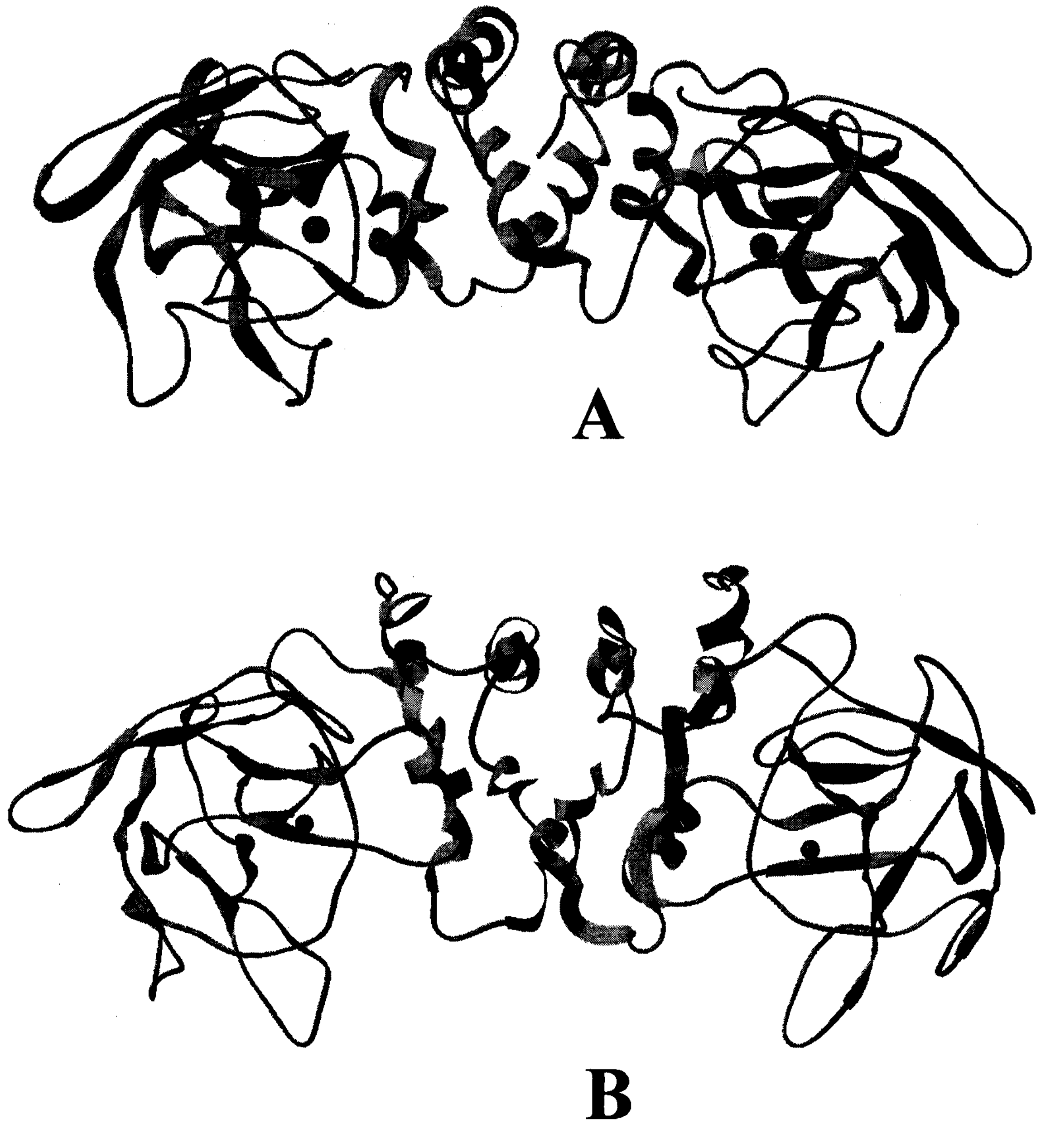

**Fig. 4:** Schematic overviews of the quaternary structures of 4-chlorocatechol 1,2-dioxygenase from *Rhodococcus opacus* 1CP (A) and hydroxyquinol 1,2-dioxygenase from *Nocardioides simplex* 3E (B).

### ACKNOWLEDGEMENTS

We thank Dr. J.R. Mason for helpful discussions. We acknowledge the financial support of the COFIN 2002 – Ministero dell'Università e della Ricerca Scientifica e Tecnologica and the Progetto Finalizzato Biotecnologie - C.N.R.; we also acknowledge the support of the European Commission RTD Programme INCO-Copernicus grant EC ICA2-CT-2000-10006.

### REFERENCES

1. R.L. Crawford and D.L. Crawford eds. *Bioremediation: Principles and Applications*, New York, Cambridge Press (1996).
2. L.Y. Young and C.E Cerniglia, *Microbial Transformation and Degradation of Toxic Organic Chemicals*, New York, Wiley-Liss (1995).
3. D.T. Gibson and R.E. Parales, *Curr. Opin. Biotechnol.*, **11**, 236 (2000).
4. T. Hudlicky, D. Gonzalez and D.T. Gibson, *Aldrichimica Acta*, **32**, 35 (1999).
5. T. Hudlicky and J.W. Reed, in *Advances in Asymmetric Synthesis,* A. Hassner, editor, JAI Press Inc. Greenwich, Connecticut (1995).
6. G.N. Sheldrake, in:. *Chirality in Industry: The Commercial Manufacture and Applications of Optically Active Compounds*, A.N. Collins, G.N. Sheldrake and J. Crosby (Eds.), John Wiley & Sons Ltd., Chichester, UK, 127 (1992).
7. D.R. Boyd, N.D. Sharma, and C.C.R. Allen, *Curr. Opin. Biotechnol.*, **12**, 564 (2001).
8. A.L. Feig and S.J. Lippard, *Chem. Rev.*, **94**, 759 (1994).
9. S. Harayama, M. Kok, and E.L. Neidle, *Annu. Rev. Microbiol.*, **46**, 565 (1992).
10. J.R. Mason and R. Cammack, *Annu. Rev. Microbiol.* **46**, 277 (1992).
11. S. Dagley, in: *The Bacteria*, J.R. Sokatch (Ed.), Academic Press, London, vol. **10**, 527 (1986).
12. W.A. Duetz, J.B. van Beilen, and B. Witholt, *Curr. Opin. Biotechnol.*, **12**, 419 (2001).
13. K.N. Timmis and D.H. Pieper, *Trends Biotechnol.*, **17**, 201 (1999).
14. B.C. Axcell and P.J. Geary, *Biochem. J.*, **136**, 927 (1973).
15. B.C. Axcell and P.J. Geary, *Biochem. J.*, **146**, 173 (1975).
16. M. Zamanian and J.R. Mason, *Biochem. J.*, **244**, 611 (1987).
17. J.R. Mason and P.J. Geary, *Meth. Enzymol.*, **188**, 134 (1990).
18. C.S. Butler and J.R. Mason, *Adv. Microbial Physiol.*, **38**, 47 (1996).
19. S. Boeren, C. Laane, and R. Hilhorst, in *Biocatalysis in Organic Media*, C. Laane, J. Tramper and L.D. Lilly (Eds.), Elsevier Science Publishers B.V. Amsterdam, (1987).
20. O. Favre-Bulle, T. Schouten, J. Kingma, and B. Witholt, *Bio/Technology* **9**, 367 (1991).
21. M. Bender (Ed.), *Interfacial Phenomena in Biological Systems*, Surfactant Science Series, Vol. 39, Marcel Dekker, Inc, New York, (1991).
22. D. Fennel Evans, and H. Wennerström, *The Colloidal Domain: Where Physics, Chemistry, Biology and*

*Technology Meet*, VCH Publishers, New York, chapter 4, (1994).

23. G.J. Salter and D.B. Kell, *Crit. Rev. Biotechnol.* **15**, 139 (1995).
24. S. D. Christian and J. F. Scamehorn (Eds.), *Solubilization in Surfactant Aggregates*, Marcel Dekker, New York, NY (1995).
25. P.C. Hiemenz and R. Rajagopalan *Principles of Colloid and Surface Chemistry*, 3 rd Edition, Marcel Dekker, Inc. New York, chapter 8, (1997).
26. R. Nagarajan, *Curr. Opin. Coll. Interface Sci.* **1**, 391 (1996).
27. F. Volkering, A.M. Breure and W.H. Rulkens, *Biodegradation* **8**, 401 (1998).
28. F. Briganti, D. Randazzo, A. Scozzafava, D. Berti, P. Baglioni, P. Di Gennaro, E. Galli and G. Bestetti, *J. Mol. Catal. B* **7**, 263 (1999).
29. D. Berti, , D. Randazzo, F. Briganti, P. Baglioni, A. Scozzafava, P. Di Gennaro, E. Galli and G. Bestetti, *J. Inorg. Biochem.* **79**, 103 (2000).
30. D. Randazzo, D. Berti, F. Briganti, A. Scozzafava, P. Baglioni. G. Bestetti, P. Di Gennaro and E. Galli, *Biotech. and Bioengin.* , 74: 240 (2001).
31. D. Berti, D. Randazzo, F. Briganti, A. Scozzafava, P. Di Gennaro, E. Galli, G. Bestetti and P. Baglioni, *Langmuir*, **18**, 6015 (2002).
32. A.E. Mars, T. Kasberg, S.R. Kaschabek, M. H. van Agteren, D.B. Janssen and W. Reineke, *J. Bacteriol.* **179**, 4530 (1997).
33. S.R. Kaschabek, T. Kasberg, D. Muller, A.E. Mars, D.B. Janssen and W. Reineke, *J. Bacteriol.* **180**, 296 (1998).
34. A.E. Mars, J. Kingma, S.R. Kaschabek, W. Reineke and D.B. Janssen, *J. Bacteriol.* **181**, 1309 (1999).
35. J. Sambrook, E.F. Fritsch and T. Maniatis, *Molecular Cloning – A Laboratory Manual*, Cold Spring Harbor Laboratory, Press Cold Spring Harbor, NY. (1989).
36. J. Sikkema, J.A.M. de Bont and B. Poolman, *J. Biol. Chem.*, **269**, 8022 (1994).
37. J. Sikkema, J.A.M. de Bont and B. Poolman, *Microbiol. Rev.*, **59**, 201 (1995).
38. B. Angelova and H.-P. Schmauder, *J. Biotechnol.*, **67**, 13 (1999).
39. G.M. Klecka and D.T. Gibson, *Appl. Env. Microbiol.*, **41**, 1159 (1981).
40. I. Bartels, H.-J. Knackmuss and W. Reineke, *Appl. Environ. Microbiol.* **47**, 500 (1984).
41. C.C.R. Allen, C.E. Walker, N.D. Sharma, N.A. Kerley, D.R. Boyd and H. Dalton, *Biocatal. Biotransf.*, **20**, 257 (2002).
42. S. Beil, J.R. Mason, K.N. Timmis and D.H. Pieper, *J. Bacteriol.*, **180**, 5520 (1998).
43. D.R. Boyd and G.N. Sheldrake, *Nat. Prod. Rep.* **15**, 309 (1998).
43. J.E.T. van Hylckama Vlieg, G.J. Poelarends, A.E. Mars and D.B. Janssen, *Curr. Opin. Microbiol.*, **3**, 257 (2000).
44. S. Fetzner, *Appl. Microbiol. Biotechnol.*, **50**, 633 (1998).
45. W. Reineke, in *Biological Degradation and Bioremediation of Toxic Chemicals*, G.R. Chaudhry ed., Portland, Oregon, Dioscorides Press, 416 (1994).
46. W. Reineke, *Annu. Rev. Microbiol.*, **52**, 287 (1998).
47. E. Dorn and H.-J. Knackmuss, *Biochem J.*, **174**, 85 (1978).

48. W. Reineke amd H.-J. Knackmuss, *Annu. Rev. Microbiol.*, **42**, 263 (1988).
49. L. Que Jr. and R.Y.N. Ho, *Chem. Rev.* **96**, 2607 (1996).
50. T.D.H. Bugg and C.J. Winfield, *Nat. Prod. Rep.* **15**, 513 (1998).
51. L. Que, Jr., in: E.C. Theil, G.L. Eichhorn and L.G. Marzilli (Eds.), *Advances in Inorganic Biochemistry*, vol. **5**: *Iron Binding Proteins without Cofactors or Sulfur Clusters*. Elsevier, New York, 167 (1983).
52. T.E. Elgren, A.M. Orville, K.A. Kelly, J.D. Lipscomb, D.H. Ohlendorf and L. Que, Jr. *Biochemistry*, **36**, 11504 (1997).
53. D.H. Ohlendorf, A.M. Orville and J.D. Lipscomb, *J. Mol. Biol.*, **244**, 586 (1994).
54. A.M. Orville, J.D. Lipscomb and D.H. Ohlendorf, *Biochemistry*, **36**, 10052 (1997).
55. F. Briganti, S. Mangani, L. Pedocchi, A. Scozzafava, L.A. Golovleva, A.P. Jadan and I.P. Solyanikova, *FEBS Lett.* **433**, 58 (1998).
56. M. Benvenuti, F. Briganti, A. Scozzafava, L.A. Golovleva, V.M. Travkin and S. Mangani, *Acta Crystallogr. D Biol. Crystallogr.* **55**, 901 (1999).
57. M. Ferraroni, M.Y. Ruiz Tarifa, F. Briganti, A. Scozzafava, S. Mangani, I.P. Solyanikova, M.P. Kolomytseva and L.A. Golovleva, *Acta Crystallogr. D Biol. Crystallogr.* **58**, 1074 (2002).
58. M. Ferraroni, M Y Ruiz Tarifa, A Scozzafava, I P. Solyanikova, M P. Kolomytseva, L.A. Golovleva and F Briganti *Acta Crystallogr. D Biol. Crystallogr.*, **59**, 188 (2003).
59. O.V. Maltseva, I.P. Solyanikova and L.A. Golovleva, *Eur. J. Biochem.* **226**, 1053 (1994).
60. O.V. Moisseeva, O.V. Belova, I.P. Solyanikova, M. Schlomann and L.A. Golovleva, *Biochemistry (Mosc)* **66**, 548 (2001).
61. M.W. Vetting and D.H. Ohlendorf, *Structure Fold Des.*, **39**, 429 (2000).
62. Manuscript in preparation.

